# Development of two surgical approaches to the pituitary gland in the Horse

**DOI:** 10.1080/01652176.2017.1415488

**Published:** 2018-01-04

**Authors:** James L. Carmalt, Brian A. Scansen

**Affiliations:** a Department of Large Animal Clinical Sciences, Western College of Veterinary Medicine, University of Saskatchewan, Saskatoon, Canada; b Department of Clinical Sciences, Colorado State University, Fort Collins, CO, USA

**Keywords:** Equine, horse, PPID, neurosurgery, interventional radiology

## Abstract

**Background:** Current treatment of equine pituitary pars intermedia dysfunction (PPID) requires daily oral medication. Minimally invasive surgical palliation of this condition is appealing as a single treatment to alleviate the clinical signs of disease, dramatically improving the welfare of the horse.

**Objective:** To develop a surgical approach to the equine pituitary gland, for subsequent treatment of PPID.

**Study design:** A cadaver study to develop methodology and a terminal procedure under anaesthesia in the most promising techniques.

**Animals and methods:** Four surgical approaches to the pituitary gland were investigated in cadaver animals. A ventral trans-basispheniodal osteotomy and a minimally invasive intravenous approach via the ventral cavernous sinus progressed to live horse trials.

**Results:** Technical complications prevented the myeloscopic and trans-sphenopalatine sinus techniques from being successful. The ventral basisphenoidal osteotomy was repeatable and has potential if an intra-operative imaging guidance system could be employed. The minimally invasive approach was repeatable, atraumatic and relatively inexpensive.

**Conclusions:** A minimally invasive surgical approach to the equine pituitary gland is possible and allows for needle placement within the target tissue. More work is necessary to determine what that treatment might be, but repeatable access to the gland has been obtained, which is a promising step.

## 1. Introduction

Pituitary pars intermedia dysfunction (PPID) or equine Cushing's disease is a common endocrine disease of the older horse. Dopamine released by hypothalamic neurons inhibits proopiomelanocortin (POMC) production in the pars intermedia of normal horses. A loss of this control leads to hypertrophy and hyperplasia of pars intermedia melanotrophs with concomitant increases in the production of POMC (Millington et al. 1988; McFarlane 2011). This 241 amino acid propeptide is subsequently cleaved into smaller peptides by prohormone convertase enzymes. These include adrenocorticotrophic hormone (ACTH1-39; 138-176 amino acid segment of POMC) as well as other POMC-derived peptides.

PPID horses with hypertrophy and hyperplasia of the pars intermedia have a grossly enlarged pituitary gland (van der Kolk et al. 2004; McFarlane 2011; Leitenbacher and Herbach 2016). The relative positioning of this region of the gland, which is sandwiched between the pars distalis and the pars nervosa, makes selective pars intermedia ablation complicated. Complete ablation of the pituitary gland would result in a multitude of other hormonal imbalances which would require daily medication, which is no better than the current standard of care treatment regime involving daily oral pergolide, a dopamine receptor agonist. While effective, the current treatment is costly due to its ongoing nature, and is labor and management intensive. Once daily medication may not appear overly onerous, however, in extensively or pasture managed horses who are infrequently handled, the need for daily medication would preclude treatment. Additionally, there is a significant emotional stress put upon the caregivers of humans and animals with chronic ongoing disease processes (Thompson and Gustafson 1996; Kelly 2014).

The preferred method of treating ACTH-dependent hyperadrenocorticism in people and dogs is the surgical ablation of hypophyseal adenomata (Meiji et al. 1998; Biller et al. 2008; Mamelak et al. 2014). Brain surgery is rarely performed in the horse. There are single case reports of the drainage of brain abscesses after localization using computed tomography (CT) (Allen et al. 1987; Cornelisse et al. 2001; Janicek et al. 2006), and a CT-guided biopsy of an intra-cerebral mass, which was subsequently diagnosed as a cholesterinic granuloma (Vanschandevijl et al. 2008). Kramer et al. (2007) reported on 3 approaches to the equine cranium (rostrotentorial, suboccipital and the transfrontal) in cadaver heads, which gave limited access to the rostral, dorsal and caudal aspects of the cerebral cortex and cerebellum. No described approach gave access to the hypophysis. There is a single case report of an open craniotomy and severing of the hypothalamic-pituitary axis using a laser (Locatelli 1984), which unfortunately does not describe the technique or outcome sufficiently well to allow repetition.

Other possible options for hypophyseal access include myeloscopy which has been described for visualization of the floor of the vertebral canal in cases of cervical vertebral malformation (Prange, Derksen, Stick, Garcia-Pereira 2011, Prange et al. 2011, 2012); a trans-sphenopalatine sinus approach modelled on the human techniques; a ventral trans-basispheniodal osteotomy; and a transcatheter approach utilizing access to the cavernous sinus of the horse originally published as a technique for pituitary effluent blood sampling (Irvine and Hunn 1984; Irvine and Alexander 1987; Alexander et al. 1994, 1996; Bons et al. 2014; Sakes et al. 2015).

The hypothesis of this study was that a surgical approach to the equine pituitary gland is feasible allowing for the further development of methods to ablate the pars intermedia. The objective of the study was to develop a novel surgical technique for access to the pituitary gland in the horse.

## 2. Materials and methods

Preliminary cadaver experiments included a myeloscopic approach in two whole cadavers and a trans-sphenopalatine sinus approach. Both were abandoned due to bleeding that impeded adequate visualization (myeloscopic approach; even in a cadaver specimen) and due to inadequate access to the caudal aspects of the sphenopalatine sinus (trans-sphenopalatine sinus approach). All animals and cadaveric parts were used under approval of the Institutional Animal Use and Care Committee of the institution in which the work was performed.

### 2.1. Ventral trans-basispheniodal osteotomy approach

Five cadaveric heads, collected immediately postmortem and frozen until use at −20 °C were used. Heads were thawed in warm water for 24 hours and subsequently drained of water before use. Heads were positioned to mimic dorsal recumbency under general anesthesia and the nose was tipped up to ensure that the base of the skull was parallel to the surgery table.

**Figure 1.**
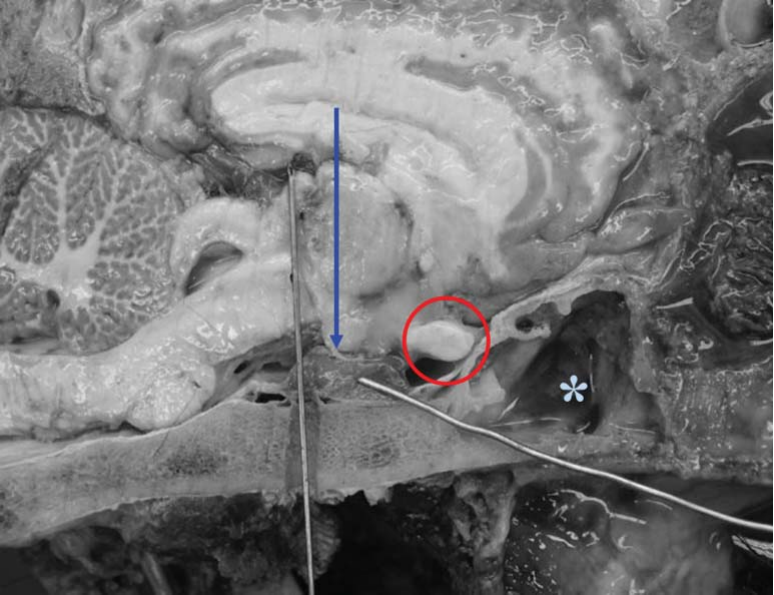
A photograph of the brain and caudal nasal passages of an equine cadaver head. Rostral is to the right and caudal to the left. The blue arrow denotes the hypothalamus, the red circle denotes the optic chiasm and the blue star illustrates the position of the sphenopalatine sinus. The oblique metal rod indicates the position of the pituitary gland while the vertical rod indicates a trans-basisphenoid approach to the gland which has been positioned too far caudally.

A flexible endoscope was advanced via the right external nares and the ventral nasal meatus into the nasopharynx. A 25W diode laser fiber (DiodeVet, Newark, DE, USA) was passed via the biopsy portal and the tissue of the dorsal pharyngeal recess was ablated using contact mode, as previously published for the treatment of guttural pouch tympany in foal (Edwards and Greet 2007). This allowed simultaneous entry into both guttural pouches and resection of the membrane separating the left and right pouches. A standard laryngotomy approach was made (Fulton et al. 2012) and a long, 12 mm wide standard laparoscopic trochar and cannula (VersaportTM Plus, Bladeless trochar, Medtronic, Minneapolis, MN, USA) was then advanced through the laryngotomy incison. The trochar was slightly withdrawn to allow the smooth edge of the cannula to sit between the corniculate processes of the arytenoid cartilages. By angling the cannula slightly rostrally while engaging the position and then moving it to a vertical position the entire larynx was displaced caudally. The sharp trochar was re-introduced and using verbal guidance from an assistant sighting midline of the horse and another checking the verticality of the cannula, in addition to using visual guidance from the flexible endoscope placed within the guttural pouch, the cannula was thrust through the roof of the nasopharynx. It was advanced until the tip of the trochar engaged the basisphenoid bone immediately rostral to the insertion of the paired longus capitis and longus capitis ventralis muscles within the guttural pouch. The trochar was removed and an auger drill bit, which had a guiding thread on the distal extent, was fed into the cannula to protect the surrounding soft tissues. A slot osteotomy was drilled through the basisphenoid bone under visual and digital radiographic guidance and then laparoscopic forceps were used to remove the final pieces of bone and confirm entry into the cranium. In this position, the pituitary gland was visible immediately under the bone, fixed to the floor of the sella turcica, and the ventral cavernous sinuses were abaxial to the drill hole (Figure 1).

Following cadaveric success, the procedure was performed in a single, live 10-year-old Quarter horse gelding under a terminal anesthetic protocol.

### 2.2. Intravenous (minimally invasive) approach

Three cadaveric heads, collected immediately postmortem and frozen until use at −20 °C were used. Heads were thawed as above, and positioned to mimic right lateral recumbency under general anesthesia. The nose was tipped up to ensure that midline of the skull was parallel to the fluoroscopy system (OEC 9900 Elite Cardiac C-Arm; GE Healthcare, Inc.; Salt Lake City, UT, USA).

**Figure 2.**
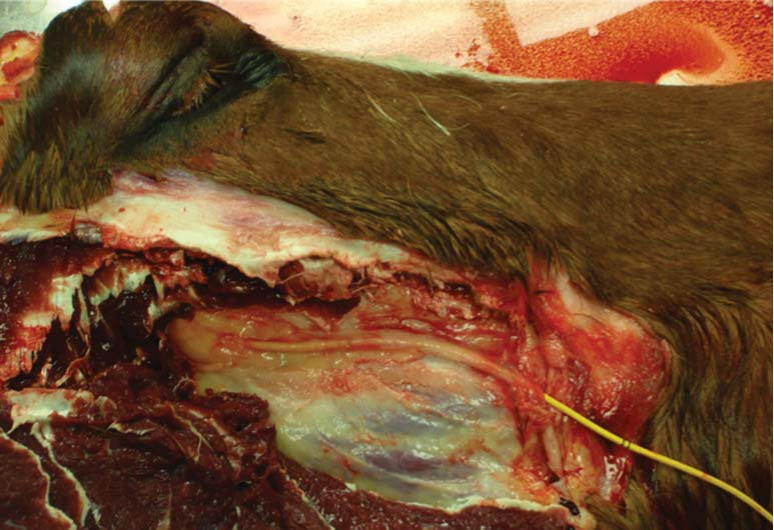
A cadaver dissection showing the catheter advanced via the deep facial vein into the orbital fissure.

**Figure 3.**
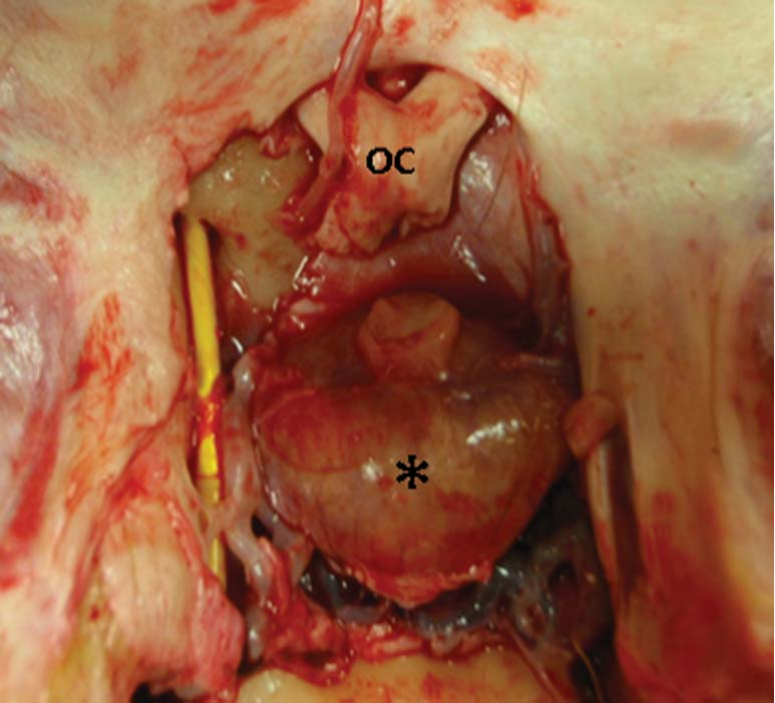
A cadaver dissection, showing the placement of the 7FG Swan-Ganz catheter in the ventral cavernous sinus of the brain having entered via the orbital fissure. Note the position of the catheter lateral to the retained pituitary gland (*). The optic chiasm is noted (OC).

As previously described by Irvine and Alexander (1987), the hair immediately rostral to the right masseter muscle mass on the lateral aspect of the face was clipped and aseptically prepared to remove surface debris and hair particles. A vertical skin incision was made through the skin overlying the deep facial vein. The sub-cutaneous tissues were carefully dissected to expose the deep facial vein, the facial artery and parotid salivary duct. The vein was isolated and elevated using a loop of 2/0 polydiaxonone suture. A #11 scalpel blade was used to make a small puncture into the vein and a 7 French (Fr) gauge (G) 110 cm pulmonary artery balloon catheter (Swan-Ganz, Edwards Lifesciences LLC, Irvine, CA, USA) was introduced (Figure 2). The catheter was advanced 25 cm and correct placement within the ventral cavernous sinus (Figure 3) was confirmed by injecting 5 mL of 240 mgI/mL Iohexol contrast solution (Omnipaque; GE Healthcare, Inc.; Princeton, NJ, USA) and visualizing the contrast column fluoroscopically in the lateral and ventrodorsal imaging planes.

**Figure 4.**
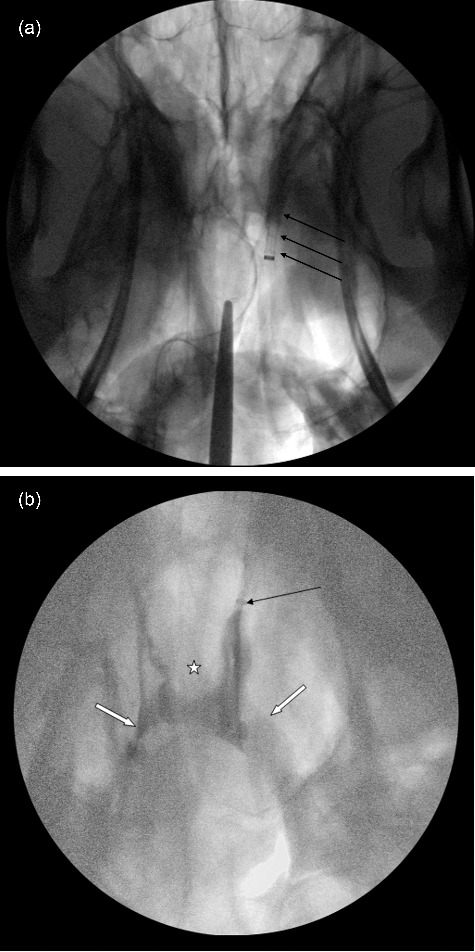
(a) A dorso-ventral fluoroscopic image of the cranium showing the catheter tip (arrows) adjacent to the pituitary gland (not visible). The caudal aspect of the pituitary gland is indicated by the tip of the scissors placed within the foramen magnum of the cadaveric head and advanced until the gland was touched. (b) A dorso-ventral fluoroscopic image of the cranium showing contrast flow in the ventral cavernous sinus around a central filling defect which represents the pituitary gland. Note the presence of the catheter (black arrow), the flow of contrast material into the emissary veins (white arrows) and the central defect representing the pituitary gland (star).

Correct entry into the cavernous sinus resulted in visible contrast flow around a large, central filling defect, which was the pituitary gland (Figure 4(a,b)).

**Figure 5.**
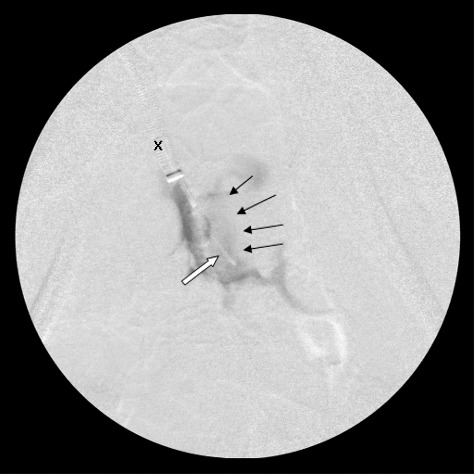
A dorso-ventral subtraction angiographic image of the cranium showing the catheter (*), needle placement within the pituitary gland (white arrow), ‘tumor blush’ (delineated by arrows) around the needle, and extra contrast material filling the ventral cavernous sinus after injection.

A 0.038” diameter, 145 cm length fixed core straight guide wire (TSF-38-145; Cook Medical, Inc.; Bloomington, IN, USA) was advanced within the Swan-Ganz catheter to the cavernous sinus under fluoroscopic guidance and the Swan-Ganz catheter was then withdrawn. A 45 cm 8Fr G angled introducer sheath (KCFW-8.0-18/38-45-RB-ANL1-HC; Cook Medical, Inc.; Bloomington, IN, USA) was advanced over the guide wire, and the tip of the sheath positioned within the cavernous sinus using intermittent administration of iohexol contrast solution and fluoroscopy to confirm location. When in the correct position, the wire guide was removed and replaced with a 56 cm 22Fr G trans-septal needle (TSNC-19-56.0 Cook Medical, Inc.; Bloomington, IN, USA). The needle was passed through the sheath and directed into the pituitary gland under fluoroscopic guidance. The gland was then injected with a combination of radiopaque contrast material and new methylene blue dye. Confirmation of gland injection was accepted when ‘tumor blush’ was seen fluoroscopically (Figure 5). Instrumentation was removed and the cranium was opened. The brain was removed leaving the pituitary gland in-situ and new methylene blue dye could be visually appreciated within the glandular tissue.

The same procedure was then performed in a live, clinically normal horse (12-year-old Quarter horse mare) and a single 26-year-old mare with hirsutism and laminitis (consistent with PPID) under terminal anesthetic procedures.

## 3. Results

It is possible that with further practice the myeloscopic technique could have been finessed, and certainly, the bleeding during endoscope placement has been previously reported (Prange et al. 2011) and overcome. The trans-sphenopalatine sinus technique using a maxillary sinus approach failed to visualize the caudal aspect of the sinus. Further, the intimate proximity of the optic chiasm relative to the pituitary gland would have rendered this technique unfeasible.

The ventral trans-basispheniodal osteotomy was reproducible and ablation of a portion (or all) of the pituitary gland was possible from this position using whichever method the surgeon might choose. After successful attempts in cadaver specimens, the procedure was performed in a single live horse under a terminal anesthetic protocol. The procedure was performed as above, however, an error in the drill position resulted in inadvertent entry into the ventral cavernous sinus and uncontrollable hemorrhage. At this point the horse was humanely euthanased by intravenous injection of Pentobarbitol (Euthanyl Forte, Bimeda-MTC Animal Health Inc., Cambridge, ON, Canada) into the jugular vein, without allowing it to recover from surgery.

In the 3 cadavers and 1 live, clinically normal, horse that underwent the minimally invasive transcatheter approach, access to the cavernous sinus and injection into the pituitary gland was possible and repeatable. Cut sections of the pituitary gland after minimally-invasive injection of methylene blue revealed intra-pituitary staining. In the PPID horse access to the cavernous sinus was achieved without complications, but at postmortem examination it was clear that while the pituitary gland was enlarged consistent with the diagnosis of PPID, the needle had been directed slightly too far, with the result that the injected methylene blue had not entered the pituitary gland, but had been deposited into the cavernous sinus blood immediately caudal to the gland.

## 4. Discussion

The treatment of pituitary tumors in humans is most often multimodal and encompasses, microsurgical, radiosurgical, radiation and medical treatments. Endonasal surgical approaches to the skull base (microscopic or endoscopic), and in particular the transsellar approach to the pituitary gland, have been well described (Joshi and Cudlip 2008; Miller et al. 2014). Despite the endoscopic approach gaining traction there is, as yet, no firm consensus on whether the microscopic or endoscopic technique is superior (Jane et al. 2005; Cappabianca et al. 2014; Kasemsiri et al. 2014). In either case, a profound knowledge of the anatomical relationships in the skull base region is required (Budu et al. 2013). Surgical access to the equine pituitary gland may allow surgical resection or chemical ablation of abnormal pars intermedia tissues, thereby controlling clinical signs of PPID in affected horses and avoiding daily oral medications. We report two surgical approaches, of which only the ventral trans-basisphenoidal osteotomy approach is completely novel. Two of the other planned approaches (i.e. myeloscopic and trans-sphenopalatine sinus approach) failed in preliminary cadaver trials.

The pituitary gland of the herbivorous quadrupeds lies ventral in the cranium within the hypophyseal fossa of the sella turcica. It is surrounded by the ventral cavernous sinus into which both pars distalis hormones and POMC-derived peptides from the pars intermedia are secreted. The comparatively long nasal passages and large paranasal sinuses of the horse, as well as the anatomical difference in the position of the hypophysis compared to humans, makes surgical access through the equine sphenopalatine sinus extremely difficult. As illustrated in Figure 1, the surgical approach is complicated by the position of the optic chiasm which lies directly between the caudal extent of the air-filled sinus and the rostral margin of the hypophysis. Even if the surgeon were to manage to position the osteotomy ventral to the optic chiasm, they would inevitably enter the rostral portion of the blood-filled ventral cavernous sinus which would result in significant hemorrhage.

The nasal passage issue has been overcome in the dog by utilizing an oral approach and computed tomographic guidance (Mamelak et al. 2014). Unfortunately, the horse mouth does not open wide enough to permit use of this technique and a modification (the ventral trans-basispheniodal osteotomy) was devised for use in the horse. It was technically demanding requiring competent use of the flexible endoscope, laser and neurosurgical skills. Accurate positioning was absolutely critical so as not to miss the hypophysis and enter the surrounding vascular sinus spaces. Use of a stereotactic frame as well as advanced image guidance such as the STEALTH neurosurgical imaging and surgical navigation system (StealthStation S7 Surgical Navigation System, Medtronic, Minneapolis, MN, USA) as used in human surgery would have greatly aided in technique development and may have improved success rates. Additional possible surgical complications are associated with damage to the vessels and nerves of the guttural pouch through which the basisphenoid bone is accessed. There are multiple reported surgical approaches to the guttural pouch. The hyovertebrotomy, also known as the Dorsal approach and Dieterich's Method, (McIlwraith and Turner 1987), access via Viborg's triangle (McIlwraith and Turner 1987), the Whitehouse and modified Whitehouse approaches, also known as the Ventral approach or the Sand's method, (McAllister 1978; Freeman 1992) and the Garm method (Garm 1946). Irrespective of the chosen approach the most common surgical complication is intractable dysphagia, which can lead to aspiration pneumonia and death. Despite this, the ventral trans-laryngeal approach to the midline of the basisphenoid bone was considered to present the least risk because of the relatively atraumatic nature of the laparoscopic cannula placement and the added benefit of soft tissue protection during the creation of the osteotomy as the drill was contained within the laparoscopic cannula. Contrary to this opinion is the fact that the ventral trans-basisphenoidal osteotomy approach (besides the described risks of inadvertent vascular or neural tissue damage) will carry a certain risk of introducing infectious agents or other contaminants to the delicate target tissues in clinical patients.

Interventional radiology is used in equine surgery for the embolization of the internal carotid, external maxillary or major palatine arteries in cases of guttural pouch mycosis. A mechanical device, such as a balloon catheter (Freeman and Donawick 1980) or thromboembolic coils (Matsuda et al. 1998; Lepage and Piccot-Crézollet 2005; Benredouane and Lepage 2012) are subsequently used to occlude the offending vessel. Similar treatment of a palatine artery pseudoaneurysm with right sided epistaxis has also been reported (McClellan et al. 2014).

The internal carotid artery passes through the guttural pouch on the caudo-medial wall, enters a sigmoid curve and then the cranium via the foramen lacerum. It then gives rise to the rostroventral infundibular (hyophyseal) arteries supplying the ventral capillary network of the median eminence of the hypothalamus (Vitums 1975). Ventral and dorsal hypophyseal vessels descend through the pars tuberalis to supply blood to the distal part of the adenohypophysis terminating in the sinusoidal network of the pars distalis. The right and left caudal infundibular arteries arise from the caudal intercarotid artery and, in some cases, the internal carotid artery, and running in a sheath of dura mater, pass through the intercavernous sinus to become a capillary network in the most distal aspect of the pars nervosa and infundibular stalk. Passage of a guide wire and catheter beyond the internal carotid artery junction with the rostroventral infundibular artery has not proven possible in our cadaveric work due to the acute angle and tortuous nature of this junction (data not shown). A transarterial approach to the pituitary gland to deliver either coils, particles (such as polyvinyl alcohol, embospheres or gelfoam) or liquid embolics (Jindal et al. 2012) into the direct arterial supply of the pars intermedia and thus spare the remainder of the gland from damage was initially envisioned, but could not be realized.

There are two paths of venous drainage; caudoventrally into the ventral petrosal sinus and foramen lacerum and secondly, rostrolaterally into the ophthalmic vein and the deep facial vein (Vitums, 1978). This latter path was exploited for long-term sampling of pituitary effluent blood (Irvine and Hunn 1984; Irvine and Alexander 1987; Alexander et al. 1996). Use of a minimally invasive transvenous approach, as outlined above, to access the pituitary gland in the horse cadavers of this report was successful and repeatable. It allowed the passage of a long flexible needle through the introducer sheath in isolated heads and in the live anaesthetized horses, under fluoroscopic guidance, to access and inject dye into the pituitary gland.

Sakes et al. (2015) published on the use of a motorized resector to partially ablate the gland using this transvenous access route, however, the future vision is that a neurolytic (such as ethylalcohol, or glycerol) or a melanotroph-specific targeting agent (Lau et al. 2015; Kaiser 2015) could be injected under guidance and result in partial ablation of the pituitary gland. In advanced stages of disease the proportion of the gland that is represented by the pars intermedia would be much greater (Leitenbacher and Herbach 2016) and thus the majority of damage caused would be directed at the offending portion of the hypophysis, however, it is likely that collateral damage to the pars nervosa or the pars distalis may also occur. The degree of lysis or ablation of the pars intermedia required to return the horse to clinical normalcy is unknown. The aim would not be to completely deprive the horse of the hormone output of the pars intermedia, but merely to disable a sufficient amount of this specific region as to improve the quality of life of these aged horses.

In conclusion, of all the surgical approaches developed to access the equine hypophysis, the transvenous approach to the ventral cavernous sinus of the cranium using interventional radiology is the least invasive. It is repeatable and, other than fluoroscopy, requires the least expensive equipment and arguably is least complicated. Further work is needed to determine which method of ablation is most effective, and how much can be ablated while preserving the critical homeostatic mechanisms of the gland. While hormone supplementation is simple and easy to use in human medicine after tumor ablation, anything other than complete resolution of clinical signs will be unsuitable in the horse because there is already an oral daily medication for PPID.
